# Emerging Trends and Hotspots in Tai Chi Fall Prevention: Analysis and Visualization

**DOI:** 10.3390/ijerph19148326

**Published:** 2022-07-07

**Authors:** Jiesi Chen, Xin Xue, Jing Xu, Jinshu Zeng, Fei Xu

**Affiliations:** 1 School of Physical Education, Hangzhou Normal University, Hangzhou 311121, China; c2021111005015@stu.hznu.edu.cn (J.C.); 20210029@hznu.edu.cn (X.X.); jinshuzeng102@gmail.com (J.Z.); 2 School of Physical Education, Nanjing Xiaozhuang University, Nanjing 211171, China; jingxu@stu.hznu.edu.cn

**Keywords:** fall prevention, tai chi, elderly, public health, bibliometric, visualization analysis

## Abstract

Recently, substantial studies have increased around the topic of the tai chi fall-prevention field. Few studies, however, have revealed the current progress and hotspots under a bibliometric analysis. Therefore, the present study aimed to conduct Citespace, a significant application for bibliometric analysis, to carry out the situation and trend in this field. This study has identified the core countries are the United States, China, Australia, and England, which are also the origins of the core institutions. Besides this, we also have found two large research groups led by Li and Sherrington. Moreover, the result has revealed that *J Aging Phys Act* and *J Am Geriatr Soc* are the primary journals. Geriatrics and gerontology, sport sciences, rehabilitation, and gerontology are the leading categories. Furthermore, one of the more important findings to come out in this study are that “elderly”, “Parkinson’s disease”, “vestibular rehabilitation”, “frail patient”, and “community fall prevention” are the research hotspots. “Women”, “proprioception”, “cognitive impairment”, “dementia”, “osteoarthritis”, and “stroke” are the potential research trend in the future. These findings suggest that the tai chi fall-prevention field has a broad research prospect. Although several questions remain uncertain currently, it is worthy for scholars to do further study.

## 1. Introduction

Falls represent a significant public health concern. Falls can lead to a general decrease in quality of life, social activity, and mobility and a large increase in fractures, head injuries, and weakness rates [1,2,3] and are also associated with an increased socioeconomic cost [4,5]. Falls have been identified as the leading cause of elderly deaths and injuries in the United States [1] as well as the second leading cause of accidental injury deaths worldwide [6]. Each year, an estimated 684,000 people die as a result of falls around the world [6]. Moreover, falls affect nearly 30% of older adults and 50% of adults over the age of 80 each year [7,8,9].

Exercise has been shown to be an effective way of lowering the risk of falling [10]. Significant evidence supports the effectiveness of balance training, functional exercise, and resistance exercise in preventing falls [9,11]. Among them, balance training is paramount, which could reduce the risk of falls by 17% [9]. Tai chi, as a form of balance training, is an exercise which aims to mobilize the body to do rotational movements continuously under a diminishing base of support [12]. Currently, tai chi is receiving increased research attention due to its efficacy in preventing falls [10,13] and fall-related abilities [14,15,16]. Moreover, tai chi is widely used to reduce the fall risks of falling in some fall-related diseases, such as Parkinson’s [17,18], vestibular disorders [19,20], and cognitive impairment (MCI) [21,22]. The effectiveness of tai chi has also been officially recognized by organizations such as the Centers for Disease Control and Prevention (CDC) [2,23] and the American Geriatric Society Clinical Practice Guidelines [23].

Overall, tai chi is a popular choice for fall prevention. In recent years, preventing falls through tai chi has received widespread concern as a research focus. There has been an increase in the number of articles on this subject with a broader research scope. Therefore, it is necessary to summarize previous research and update the current frontiers of this field. Unfortunately, most articles have focused on the preventive effect of tai chi on falls or fall-related diseases, and few have concentrated on the current state and evolution of this field. This makes it difficult to discover hotspots for thousands of papers. Bibliometric analysis is based on multiple indexes, such as references, authors, journals, countries, and institutions, and it can assist scholars in identifying problems and hotspots in current research [24]. Citespace is a significant application for bibliometric analysis, which is able to label a specialty and detect emerging trends and sudden changes [25]. Thus, we conducted Citespace to visualize the research trend and hotspots of this field in order to assist scholars worldwide in better understanding the current situation and frontiers. Furthermore, to the best of our knowledge, this is the first study to use bibliometric analysis to investigate the overall progress of the tai chi fall prevention.

## 2. Materials and Methods

Since fall-prevention research involves multiple disciplines [26], we needed a comprehensive database to collect the literature. *Web of Science Core Collection* (*WoSCC*) has a sufficient number of high-quality and high-impact journals, which is suitable for our study to investigate complete and accurate literature data. Therefore, the data of this study were derived from *WoSCC*. The search and download date was 31 December 2021.

**Figure 1:**
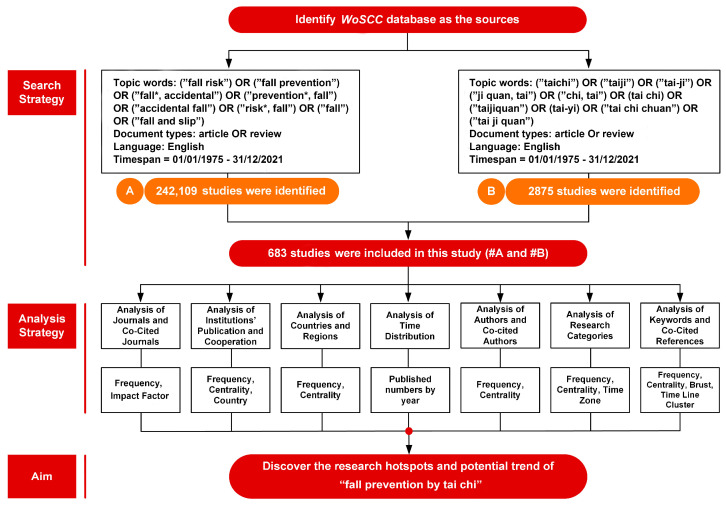
The flowchart of the research design.

We started by reading the article titles and abstracts to see if they were relevant to the tai chi fall-prevention research. We included studies in this bibliometric analysis if they met the following criteria: (1) tai chi fall topic; (2) investigation of the effects of tai chi on various people’s fall prevention, such as fall risk, fall prevention, accidental fall, and so on; and (3) different indicators, exercise modalities, and protective measures and neurological, physiological, and psychological mechanisms of tai chi fall prevention. Exclusion criteria included (1) incompatibility with the tai chi fall-prevention topic; (2) tai chi not featured as the primary intervention; (3) an emphasis on tai-chi-related philosophy, arts and humanities, music, formal aesthetics, and meditation; and (d) no inclusion of health indicators. Using these criteria, we eventually included 683 articles and reviews and downloaded all the articles’ contents in text format, which we then entered into Citespace. Our search criteria were as follows: topic words = ((“Fall Risk”) OR (“Fall Prevention”) OR (“Fall*, Accidental”) OR (“Prevention*, Fall”) OR (“Accidental Fall”) OR (“Risk*,Fall”) OR (“Fall”) OR (“Fall and Slip”)) AND ((“Taichi”) OR (“Taiji”) OR (“Tai-ji”) OR (“Ji Quan, Tai”) OR (“Chi, Tai”) OR (“Tai Chi”) OR (“Taijiquan”) OR (“Tai-yi”) OR (“Tai Chi Chuan”) OR (“Tai Ji Quan”)). Timespan = 01/01/1975–31/12/2021. Document type = article and review. Language = English (the specific research design and search process refer to Figure 1 and Table S1, respectively).

We used Citespace visualization software (V.5.5.R2, 64 bit) to analyze the collected records. The advantages of this method are that it can identify research frontiers and potential trends visually. Before analysis, we needed to set the time slicing, threshold, pruning, and node type, etc. Firstly, based on the previous retrieved result, we found that the first study in this field appeared in 2006. Therefore, we set the timespan from 2006 to 2021. Other parameters set were as follows: year per slice = 1, term source = title, abstract, author keywords and keywords plus, scope = within slices, strength = cosine, selection criteria = top 50, and pruning parameter = Pathfinder.

Then we selected the nodes about “author”, “country”, “institution”, “keyword”, “research category”, “co-cited reference”, “co-cited author”, and “co-cited journal” to analyze the specific research status. The indicators for analyzing these nodes were mainly adopted frequency, centrality, burst detection, and cluster. All of these are the essential components of bibliometric analysis and have the following characteristics, respectively. Frequency usually refers to the node’s publication numbers, frequency of occurrence, and citation counts. Among them, citation count is related to the research value. High co-cited references almost constitute the knowledge structure in this field. Centrality means the node’s percentage of their shortest path numbers in the network. A node with high centrality means that it has the most connections to other fields [25]. In the visualization map, the higher the frequency of the node, the larger the size of the node, and the higher the centrality of the node, the larger the purple ring around the node. Burst detection detects the emerging discoveries and scientific breakthroughs, and it can also provoke researchers to study themes from new perspectives [25]. Clusters are mainly used to identify the hotspots. The importance of each cluster is listed in descending order. In addition, time zone and timeline are important visual types in Citespace, which are used to visually analyze the evolution of a specific node type from the time, which goes far to near (left to right) [25]. In this way, we analyzed the collected 683 records and determined the cooperative relationships, hotspots, and potential frontiers in the tai chi fall-prevention field.

## 3. Results

### 3.1. Time Distribution

**Figure 2:**
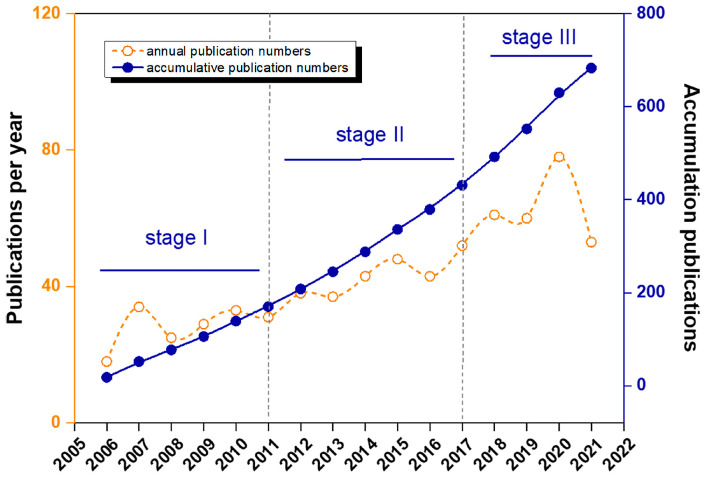
Annual and cumulative distribution of publications trend map.

Annual and accumulative publication numbers are important indicators to judge the change of research attention. The time distribution of publication on this field has shown in Figure 2. As we can see, 2006 was the early part of this field, with 683 studies published. Year after year, the number of annual publications increased and reached a high of 78 studies in 2020. According to the accumulated publication figures, the growth rate increased rapidly. It is clear that research interest is continuing to rise.

### 3.2. Analysis of Countries and Regions

**Figure 3:**
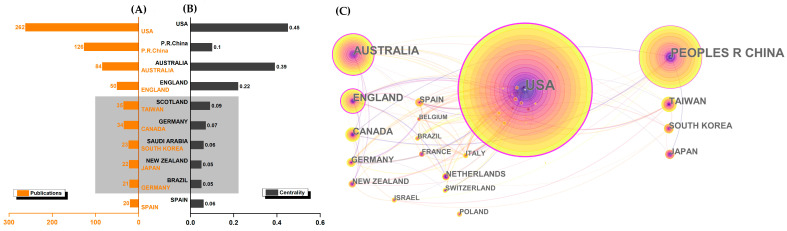
Visualized map of the leading countries and regions. (**A**) Top ten countries and regions by publications; (**B**) top ten countries and regions by centrality; (**C**) cooperation map of countries and regions.

As shown in Figure 3, the United States (262) has the highest amounts of publications, whereas China (126) ranked second, Australia (84) ranked third, and England (50) ranked fourth, respectively. Furthermore, the United States (0.45) also has the highest centrality. Australia (0.39), England (0.22), and China (0.1) ranked second to fourth, respectively. These four countries have made large contributions. Interestingly, some countries’ publications did not match their centrality. Scotland, for example, has a high centrality, but its publications have not ranked in the top ten. The partnerships of many countries and regions were relatively close (Figure 3C), which were generated by Citespace into 48 nodes and 148 links.

### 3.3. Analysis of Institutions

**Figure 4:**
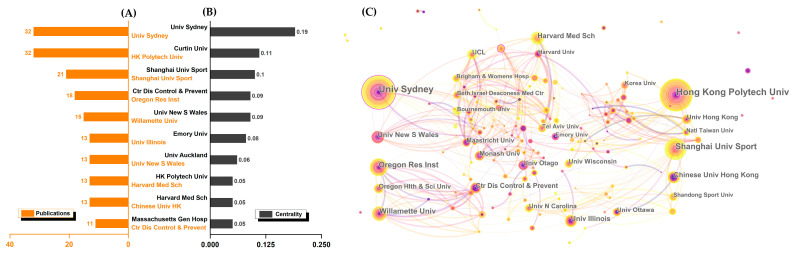
Visualized map of the leading countries and regions. (**A**) Top ten institutions by publications; (**B**) top ten institutions by centrality; (**C**) collaboration map of institutions.

Univ Sydney (32), Hong Kong Polytech Univ (32), and Shanghai Univ Sport (21) have the highest publication numbers (Figure 4). Univ Sydney (0.19), Curtin Univ (0.11), and Shanghai Univ Sport (0.10) also have the highest centrality. Notably, Univ Sydney ranked first in both the publication and centrality ranking. These institutions were of great significance in promoting research. In addition, the cooperation between many institutions was relatively close, and the network map was plotted by Citespace with 1017 nodes and 2434 links. Surprisingly, the institutional collaborations were regional. There were few links between institutions on different continents.

### 3.4. Analysis of Journals and Co-Cited Journals

**Figure 5:**
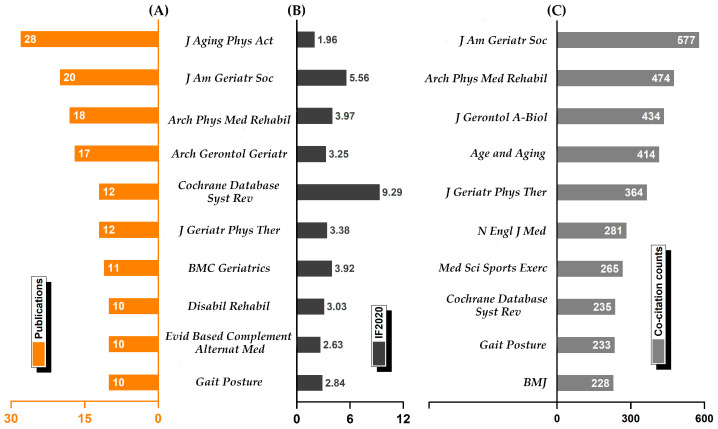
Visualized map of the leading journals. (**A**) Top ten journals by publications; (**B**) top ten journals by impact factor (IF) in 2020; (**C**) top ten co-cited journals based on citation counts. Note: The *WoS* provided the publications and impact factor (IF), and Citespace provided the citation counts.

According to the results of the journal analysis (Figure 5)*, J Aging Phys Act* (28 studies, IF = 1.961) has the most publications. *J Am Geriatr Soc* (20, IF = 5.562), *Arch Phys Med Rehabil* (18, IF 2020 = 3.966), and *Arch Gerontol Geriatr* (17, IF = 3.25) followed. These four journals were more concerned with this topic. Furthermore, with the exception of *Cochrane Database Syst Rev* (12, IF = 9.289), the impact factors of the top ten journals ranged from 1.96 to 5.56. In addition, *J Am Geriatr Soc* (577) has the most co-citation counts, followed by *Arch Phys Med Rehabil* (474), *J Gerontol A—Biol* (434), and *Age and Aging* (414).

### 3.5. Analysis of Authors and Co-Cited Authors

**Figure 6:**
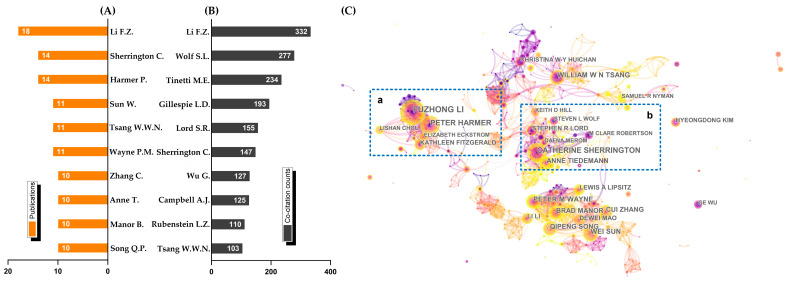
Visualized map of the leading authors and co-cited authors. (**A**) Top ten authors by publications; (**B**) top ten authors by co-citation counts; (**C**) collaboration map of authors.

According to the Figure 6, Li, Sherrington, Harmer, and Wolf appear to have had a significant impact on the advancement of tai chi fall-prevention research. In addition, Citespace calculations showed that 2666 authors contributed to this research area, with a total of 8101 connections (data not presented in the collaboration map). Furthermore, two large research groups were identified though there were few collaborations between the two groups, which were led by Li (group a) and Sherrington (group b), respectively.

### 3.6. Analysis of Research Categories

**Figure 7:**
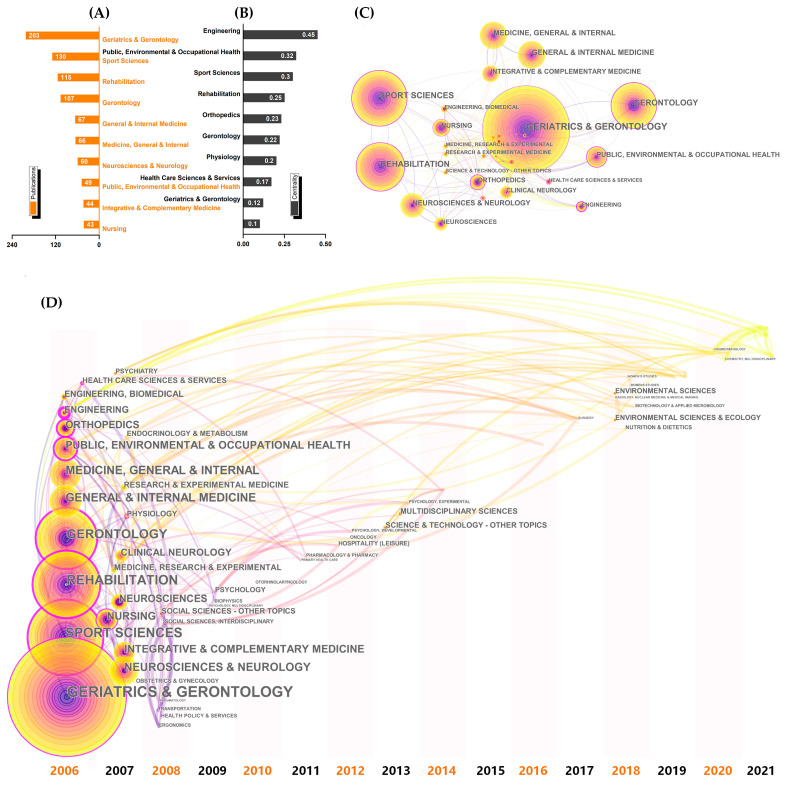
Visualized map of the research categories. (**A**) Top ten research categories by publications; (**B**) top ten research categories by centrality; (**C**) collaboration map of research categories; (**D**) time zone view of research categories.

The research category map, which has 80 nodes and 219 links, demonstrates that tai chi fall-prevention research is a multidisciplinary and comprehensive research field (Figure 7). Geriatrics and gerontology (203 studies) and gerontology (107) have a high number of publications, ranking first and fourth, respectively. Sport sciences (130) and rehabilitation (115) were ranked second and third, respectively. Engineering (0.45); public, environmental, and occupational health (0.32); and sport sciences (0.30) have high centrality, which played an important role in this field.

Figure 7D shows that a number of research categories emerged in 2006 and 2007, including geriatrics and gerontology, gerontology, sports science, social science, rehabilitation, neurosciences and neurology, and obstetrics and gynecology. Around 2012, new fields such as psychology, oncology, and otolaryngology emerged. In addition, chemical pathology, environmental sciences, nutrition and dietetics, and biotechnology and applied microbiology have been emerging categories in recent years.

### 3.7. Analysis of Keywords

**Figure 8:**
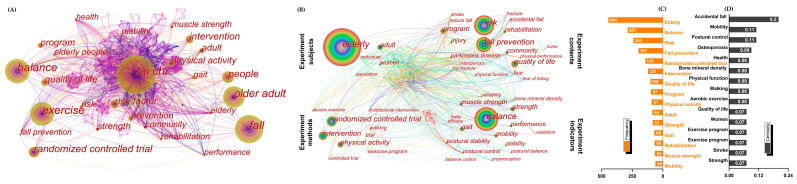
Visualized map of the keywords. (**A**) Original keywords map; (**B**) modified keywords map. Network: N = 271, E = 1291; (C) top ten keywords by publications; (**D**) top ten keywords by centrality.

Because the original visualized map (Figure 8A) was disorganized, we merged some repetitive and synonymous keywords (refer to Table S2) and hid “tai ji”, “fall”, “people”, “health”, and “exercise”, as shown in Figure 8B. The four primary categories are more clearly presented: (1) The main experiment subjects were “elderly” and “women”; (2) the main experiment contents were “fall prevention”, “fear”, “hip fracture”, “accidental fall”, “risk”, “Parkinson’s disease”, “osteoporosis”, and “quality of life”; (3) the main experiment methods were “randomized controlled trial”, “multifactorial intervention”, and “controlled trail”; and (4) the main experiment indicators were “balance”, “postural stability”, “gait”, “muscle strength”, “bone mineral density”, “mobility”, and “proprioception”. Among them, “elderly” (444), “balance” (287), and “risk” (240) were frequently used keywords (Figure 8C), while “accidental fall” (0.20), “mobility” (0.11), and “postural control” (0.11) have high centrality (Figure 8D).

**Figure 9:**
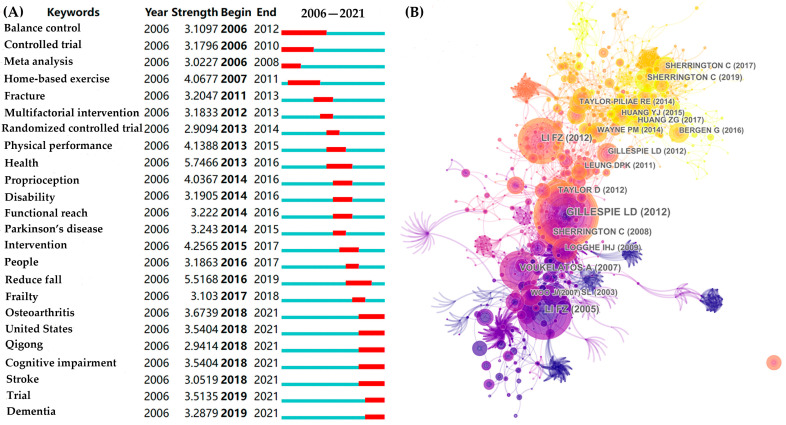
(**A**) Visualized map of burst keywords; (**B**) visualized map of co-cited reference.

The burst keywords are depicted in Figure 9A. “Balance control” and “controlled trial” have long burst durations. “Health” and “reduce fall” have strong burst intensions. Notably, “osteoarthritis,” “qigong,” “cognitive impairment,” “stroke,” and “dementia” were burst keywords in recent years (began in 2018–2019).

### 3.8. Analysis of Co-Cited References

Figure 9B has shown the core reference in this field. Table 1 and Table 2 show the top 10 co-cited references with high citation counts or centrality. As we can see, the top co-cited and centrality reference was authored by Gillespie. Furthermore, Li’s (53, 45) and Sherrington’s (31, 24, 23) articles also have high citation counts. Hackney’s (0.19) and Ashburn’s (0.17) articles have high centrality. All these articles are the core articles and have an important basis for promoting the development of this field.

**Figure 10:**
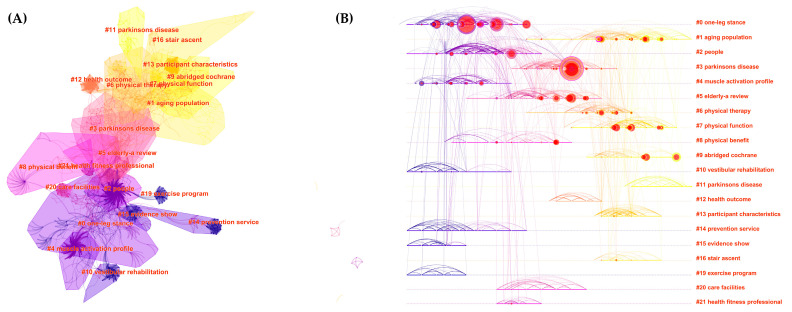
Visualized map of the co-cited reference clusters and timeline. (**A**) Map of the co-cited references clusters; (**B**) timeline view of co-cited references clusters.

Figure 10 shows a total of 20 clusters (the specific silhouette of each cluster refers to Table S3). Among them, “One-leg stance” (#0) was the most important cluster, which also has the most frequent occurrences in Table 1, followed by “Aging population” (#1), “People” (#2), “Parkinson’s disease” (#3), and “Muscle activation profile” (#4). According to the timeline view, “Aging population” (#1) has the longest attention duration, while “Parkinson’s disease” appeared twice (#3, #11) in different time periods. It implied that they have received more attention in this field.

## 4. Discussion

Overall, the research in this area is a broad research prospect with a clear upward trend of publications. The first research was concerned in 2006 and reached a peak in 2020 (Figure 2). Its development trend can be divided into three stages: the initial stage (2006–2011, 18–34), subsequent development stage (2012–2017, 37–52), and rapid development (2018–2021, 53–78) stage, which each resulted in an additional 28, 15, and 20 averages, respectively, more than the previous phase.

The core countries were the United States, China, Australia, and England. We deduced that their leading edge could have the following connections. The typical institutions of the United States may provide a solid foundation for conducting research in the tai chi fall-prevention field. Harvard Medical School, for example, was the most visible burst institution in older adults’ accidental falls [26], and Harvard University has the most publications and greatest centrality in tai chi research [37]. Following that, because tai chi originated in China and has played an important role in traditional Chinese sports, it has the potential to provide China with a wealth of research resources. Australia and England, like the United States, have some typical institutions focusing on related fields. The University of Sydney (Australia) has the most publications, and the University of Oxford (England) is the most important institution in the study of accidental falls in older adults [26]. Furthermore, we considered the possibility of a corresponding relationship between scientific research and the economy. It is well-understood that the state of each country’s economy greatly influences the amount of funding that research institutions receive for basic and applied research and development (R&D), and research funding sows the seeds for future education, science, and technologies. According to the 2020 global GDP ranking, the United States is first, China is second, England is fifth, and Australia is thirteenth. This, we believe, is a critical factor that must not be overlooked. Interestingly but not surprisingly, some countries have high centrality but publish less (Figure 3B). Their publications are of high quality and frequently make transformative discoveries [38].

Based on the strength of the links, we observed that Western countries have stronger international relations than Eastern countries (Figure 3C). In parallel, they were more concentrated on this research field. We believe this is due, in large part, to the importance these countries place on public health issues. Fall is a major functional public health issue in the Western European region. Furthermore, Western Europe has the highest rate of fall-related injuries and mortality among the elderly [39]. In 2017, nearly 11.7 million elderly quested medical attention for an injury, with 8.4 million requiring assistance due to a fall [39]. Therefore, it is not surprising that some high-quality research on fall-prevention methods (e.g., tai chi) has emerged in Western Europe.

Similarly, the top ten institutions came from the United States, China, and Australia. The four core institutions were Univ Sydney, Shanghai Univ Sport, HK Polytech Univ, and Curtin Univ. Among them, the Univ Sydney was the most prolific institution and has the highest centrality. It could be because the Univ Sydney has the most publications on accidental falls in the elderly [39]. For Shanghai Univ Sport, the main contributing factor may be its multiple research areas, which include postural control, physical-related factors, functional exercise screening, and ankle neuromuscular function. HK Polytech Univ appears to be focused on intervening in fall-related diseases such as stroke patients, elderly with visual impairment, and Parkinson’s patients, which were the current concerns. Interestingly, we observed that Curtin Univ has a high centrality, and their research may lead to significant influence. This institution’s most cited article (a review) focused on the effects of interventions on reducing elderly falls [40]. With unexpected findings, it is uncertain that exercise could reduce fall risks in care facilities as well as that additional physiotherapy and bed sensor alarms could reduce falls in hospitals. This result undoubtedly draws attention in most situations where tai chi has a positive effect on fall-related factors. Notably, the visualized map of institutional partnerships (Figure 4C) showed that the four core institutions have almost no connection except the weak link between Curtin Univ and HK Polytech Univ. Furthermore, the inter-institutional collaborations are regional in nature. In a nutshell, there was a clear continental cooperative division. America’s, Australia’s, and Asia’s institutions have close relationships with their respective continental institutions, but intercontinental cooperation is limited.

It is possible to screen core journals by analyzing journal publication numbers and citation counts of co-cited journals. Almost all of the top ten journals, according to our findings, are related to geriatrics, sport science, or rehabilitation. *J Aging Phys Act, J Am Geriatr Soc, Arch Phys Med Rehabil,* and *J Gerontol A—Biol* were the primary journals. These journals are concerned with the elderly’s health promotion and cover a wide range of research topics, which are also recommended for subsequent author submissions. *J Am Geriatr Soc* focuses on clinical nursing for the elderly. *Arch Phys Med Rehabil* conducts rehabilitation medicine research, focusing on the effects of physical and pharmaco-therapies on patients with disabilities and chronic diseases. *J Aging Phys Act* is interested in researching the relationship between physical activity and the aging process. *Arch Gerontol Geriatr* focuses on gerontology topics such as experimental gerontology, clinical gerontology, and social gerontology. In addition, the journal with the highest impact factor (IF 2020 = 9.289), *Cochrane Database Syst Rev*, was notable. Gillespie L.D., a well-known author, published a study in this journal that was co-cited 69 times and cited 496 times [27], which drew a great deal of attention from researchers.

We identified the core group and potential collaborations by analyzing the authors’ publications and co-citations and visualizing the cooperation map. As we can see, the leading authors were Li (Oregon Res Inst), Sherrington (Univ Sydney), Harmer (Willamette University), and Wolf (Emory Univ). They are not only prolific in terms of publications and co-citations but also well-known within their respective affiliates. Actually, almost all of the institutions to which they each belong are leading institutions (see Figure 4). It is widely acknowledged that exceptional institutions and outstanding scholars complement and supplement one another. Meanwhile, their ability to catch hotspots also contributed to their success. Li (researched fall-related diseases) and Wolf (investigated community-based fall prevention), for example, are both fall-prevention professionals who caught the tai chi fall-prevention research hotspots currently (see Figure 8 and Figure 9). As a result, those authors have high co-citations and are among the leading scholars. Moreover, we also cannot ignore the outstanding scholars’ complementarity and cooperation. Hammer has worked closely with Li and is the co-author of 12 articles in this field. Aside from that, we noticed two large research groups led by Li (group a) and Sherrington (group b). Group a focused on disease intervention, clinical treatment, multi-exercise intervention, and cognition impact. Li et al., for example, used tai chi to improve balance and reduce fall risks in people with mild-to-moderate Parkinson’s disease [18]. Group b was more concerned with fall prevention. Balance-challenging exercise, according to Sherrington et al., has a significant impact on preventing elderly falls [9]. These two groups concentrated on different aspects of tai chi fall prevention. However, collaboration between the two groups was rare.

According to the categories and burst keywords visualization results (Figure 7 and Figure 9), we could find the evolution of research directions surprisingly. Geriatrics and gerontology, gerontology, sport sciences, and rehabilitation were the primary research areas. Early research is instructive, and many studies investigated the effect of tai chi on functional ability and health-related quality of life in high-risk elderly [41,42]. At that time, tai chi was mostly regarded as a home-based exercise. In subsequent studies, more research was conducted to investigate tai chi’s therapeutic/rehabilitative and preventive effects for issues such as Parkinson’s disease [18], stroke [43], and osteoarthritis [44]. The research methods of “multifactorial intervention” and “randomized controlled trials” were also increasingly concerned. During this period, the research became more practical. Recent studies in comparison to previous ones have gradually tended to concentrate on the mind and body of patients with multiple sclerosis [45] and knee arthritis [46] as well as the verbal working memory in elderly with intellectually disabilities [47] and cognitive impairment [22]. In terms of research content, recent research has involved many disciplines, including medicinal, chemistry, pharmacology and pharmacy, genetics and heredity, neurosciences and neurology, psychiatry, and rehabilitation. Therefore, there has emerged a multidisciplinary and comprehensive research field with enriching categories. Furthermore, in terms of time zone (Figure 7D), chemical pathology, environmental sciences, nutrition and dietetics, and biotechnology and applied microbiology were the emerging categories with the potential to become hotspots in the future research.

The analysis of references is also an important way for us to grasp the research hotspots. According to Table 1 and Table 2, the research designs of these articles were mainly about randomized controlled trials (RCTs) [10,18,28,29,30,31,34,36] and reviews (meta-analyses [9,32,35]) [27,33]. Therefore, these two designs are the prioritized choices for scholars writing high-quality articles. Furthermore, most articles were aimed at preventing elderly falls (in the community). In terms of fall-related diseases articles, they were more focused on the frail and on Parkinson’s patients. Therefore, “elderly”, “community”, “frail”, and “Parkinson’s patients” were the current or potential hotspots for scholars, which should be gaining a great deal of attention.

In order to better understand the hotspots and future research directions, based on the main keywords (Figure 8 and Figure 9) and clusters results (Figure 10 and Table 1 and Table 2), we summarized four primary themes of this field (specific summarized keywords and clusters refer to Table S4):

i. Targeted therapy. Tai chi is a movement that has been used in a variety of clinical settings to improve flexibility, coordination, exteroception, and proprioception [48,49]. Its movement features may have targeted and displayed potentially therapeutic effects for a wide range of fall-related diseases falls. Currently, tai chi is widely discussed for preventing falls in “Parkinson’s disease”, “vestibular rehabilitation”, “fracture”, “osteoarthritis”, and “stroke”. Numerous studies have shown that tai chi improves these patients’ balance abilities [18,19,20,44,50] while also lowering their risk of fracture [51]. It appears to be an accepted conclusion though the mechanism underlying the therapeutic changes in participants’ motor control and mobility remains unknown [18]. In addition, there are some ambiguous research findings that merit further investigation. Scholars have long debated the efficacy of tai chi in preventing falls in frail patients. Many academics believe that tai chi can assist transitionally frail patients in avoiding falls [36,52] and reducing their fear of falling [42]. However, a small number of studies have claimed that tai chi is not significantly effective in preventing falls in frail patients [31,36]. Furthermore, previous studies on the effect of tai chi on osteoporosis patients were inconsistent, and some studies show that tai chi improves bone density, which can help people with osteoporosis avoid falls [53, 54, 55], while Lee and his colleagues disagreed [56]. Further studies with more concerns in this direction are therefore suggested.
ii. Community fall prevention. Every year, at least one-third of community-dwelling people over the age of 65 fall, imposing a significant burden on public health [57]. Tai chi is a vital, community-based exercise that is not limited by location or age. Numerous studies have shown that tai chi improves balance ability [27,58,59]. There has been a great deal of concern about preventing community-dwelling people from falling. Currently, the focus of research appears to be shifting from a single tai chi intervention [60,61] to a multifactorial intervention (tai chi combined with cognitive–behavioral strategies or usual care) [62,63] as well as the effect of a specific tai chi movement [64,65]. Numerous studies have found strong evidence that tai chi can reduce falls among community-dwelling people [9,10,11,27,28,32]. However, some studies contradict this finding (Table 1 and Table 2) [29,30,66]. The ineffective results could be attributed to relatively high withdrawal and adherence rates [67] as well as low exercise intensity [29]. Furthermore, some researchers have also focused on community-based caregivers [68,69]. During the tai chi intervention, caregivers could safely manage people, improve their exercise commitment, and tailor their nursing interventions [16]. It has played an important role in preventing falls in the community, and it is a research direction that scholars should not overlook.
iii. Research paradigm. The research paradigm had three components: participant, indicator, and design. According to participants, the elderly are the most vulnerable to falls and the most frequent tai chi practitioners. Tai chi, with its slow movements and emphasis on balance, is an appropriate exercise for the elderly to prevent falls [10,11,27,30,31,32,33,35,36,66] and reduce the risk of falls in related diseases [18,21,22,31,36,62,70]. Given the growing number of the elderly, more research on this population is required to clarify the accuracy of the intervention effect. In addition, female participants are a new focus that has emerged in recent years (Figure 7D). Recently, researchers have concentrated on bone mineral density, knee and ankle proprioception, and multiple sclerosis in postmenopausal women [55,71,72,73]. Thus, the scope of this research should be expanded. In terms of indicators, the most common indicator is “one-leg stance.” Certain movements are associated with “one-leg stance”, which assists in the practitioner’s physical stability (challenging balance, requiring individuals to move with less support) [74]. Furthermore, balance and postural control [75,76], gait [43], muscle activation profile [77], strength [78], bone mineral density [55], and proprioception [72] are all important indicators. Overall, tai chi has been shown to improve these functions, which may play an important role in fall prevention. However, there has been little research on the “muscle activation profile”. Furthermore, “proprioception” has a high burst intension (Figure 9A), indicating that it merits more attention. For research designs, randomized controlled trials (RCTs) [79] and meta-analysis [80] are important types of research that scholars prefer when selecting references (Table 1 and Table 2). This suggests that rigorously designed experiments and high-quality quantitative reviews are more conducive to producing high-quality research articles. These types of articles are more likely to be read and cited by scholars. However, no study appears to have precisely defined the style, timing, and intensity of tai chi fall-prevention research. There is also no widely accepted standard in this area. This, we believe, would be an important direction for future research.
iv. Psychological factors. Tai chi is also used to prevent falls in people with cognitive impairment [21,22,62,70] and dementia [63,81,82], which have recently been identified as hotspots. According to our findings, tai chi appears to be a new option for reducing the incidence of MCI progression to dementia [16]. Tai chi exercise may be related to specific task requirements such as movement recall, movement planning and switching, and visuomotor processing, implying that tai chi is beneficial to executive function [22]. As a result, further research should be conducted to investigate the impact of tai chi on the specific cognitive function of patients with cognitive impairment.

There are some limitations that should be considered form our study. First, Citespace software is primarily based on the *WoS* database and currently does not integrate with other databases, such as *PubMed, Scopus, Springer, EBSCOhost*, and *ProQuest*. Second, we only included literature in English. It is possible that high-quality literature with original ideas and marginal research was overlooked. As a result, our findings should be interpreted cautiously, at least in part.

## 5. Conclusions

In order to summarize the current research situation and frontiers of the tai chi fall-prevention field, we conducted a systematically bibliometric analysis of 683 articles from *WoSCC* by using Citespace.

Firstly, we discovered the research trend by analyzing the time distributions. We found that this field has a good research prospect, and the development trend could be divided into the initial stage (2006–2011), subsequent development (2012–2017), and rapid development stages (2018–2021).

Secondly, the results in this study also revealed the primary countries (the United States, China, Australia, and England), institutions (Univ Sydney, Shanghai Univ Sport, HK Polytech Univ, and Curtin Univ), authors (Li, Sherrington, Harmer, Wolf), and journals (*J Aging Phys Act, J Am Geriatr Soc, Arch Phys Med Rehabil*, and *J Gerontol A—Biol*) that were of great significance to promoting research in this field. Particularly important were the United States, Univ Sydney, and Li because all of them have the highest publications and centrality, respectively.

Thirdly, in terms of cooperation, we identified that Western countries are more concerned about this field and have closer international relations than Eastern countries. For institutions, the relationships of institutions are regional. America, Australia, and Asia’s institutions have little intercontinental cooperation. Moreover, we also found two large research groups led by Li and Sherrington.

In terms of categories, the results showed that this field is a diversified and comprehensive field with various categories. Among them, geriatrics and gerontology, sport sciences, and rehabilitation are the main categories, while chemical pathology, environmental sciences, nutrition and dietetics, and biotechnology and applied microbiology are the emerging categories. They may become the hotspot areas in future research.

Furthermore, the research hotspots and potential trends are the most important findings of this study. “Parkinson’s disease”, “vestibular rehabilitation”, “frail patient”, “community fall prevention”, and “nursing practice” are currently hotspots. The “elderly” are the most concerned. “RCT” and “meta-analysis” are important study designs. Future research trends include “women”, “proprioception”, “cognitive impairment”, “dementia”, “osteoarthritis”, and “stroke.” Overall, tai chi is gaining popularity as a fall-prevention technique. This study suggests some potential future research areas.

## Figures and Tables

**Table 1:** Top ten co-cited references with high citation counts.

Rank	Co-Cited References	Citation	Cluster	Year	Author
1	Interventions for preventing falls in older people living in the community [27]	65	3	2012	Gillespie L.D.
2	Tai chi and fall reductions in older adults: a randomized controlled trial [10]	53	0	2005	Li F.Z.
3	Tai Chi and Postural Stability in Patients with Parkinson’s Disease [18]	45	3	2012	Li F.Z.
4	A randomized, controlled trial of tai chi for the prevention of falls: The central Sydney tai chi trial [28]	39	0	2007	Voukelatos A.
5	Effective Exercise for the Prevention of Falls: A Systematic Review and Meta-Analysis [9]	31	2	2008	Sherrington C.
6	Effectiveness of Tai Chi as a Community-Based Falls Prevention Intervention: A Randomized Controlled Trial [29]	28	5	2012	Taylor D.
7	Lack of Effect of Tai Chi Chuan in Preventing Falls in Elderly People Living at Home: A Randomized Clinical Trial [30]	25	0	2009	Logghe I.H.J.
8	Intense tai chi exercise training and fall occurrences in older, transitionally frail adults: a randomized, controlled trial [31]	24	0	2003	Wolf S.L.
9	Exercise to prevent falls in older adults: an updated systematic review and meta-analysis [32]	24	9	2017	Sherrington C.
10	Exercise for preventing falls in older people living in the community [11]	23	9	2019	Sherrington C.

**Table 2:** Top six co-cited references with high centrality (centrality ≥ 0.1).

**Rank**	**Co-Cited References**	**Citation**	**Centrality**	**Year**	**Author**
1	Interventions for preventing falls in older people living in the community [27]	0.23	1	2012	Gillespie L.D.
2	Impact of Tai Chi Chu’an practice on balance and mobility in older adults: an integrative review of 20 years of research [33]	0.19	3	2005	Hackney M.E.
3	A randomised controlled trial of a home-based exercise programme to reduce the risk of falling among people with Parkinson’s disease [34]	0.17	2	2012	Ashburn A.
4	The effect of fall prevention exercise programmes on fall induced injuries in community dwelling older adults: systematic review and meta-analysis of randomised controlled trials [35]	0.12	3	2007	EL-Khoury F.
5	Training Program on Postural Control and Walking Ability in Older People [35]	0.11	0	2008	Lelard T.
6	Effects of exercise programs on falls and mobility in frail and pre-frail older adults: A multicenter randomized controlled trial [36]	0.10	5	2012	Faber M.J.

## Data Availability

The data presented in this study are available in supplementary material.

## Supplementary Materials

The following supporting information can be downloaded at: https://www.mdpi.com/article/10.3390/ijerph19148326/s1, Table S1: Table of the specific search process, Table S2: Table of the Merged Keywords, Table S3: Ranking of co-cited reference cluster, Table S4: The primary themes of Tai Chi fall prevention field.
