# Supplementary material for: Emerging Trends and Hotspots in Tai Chi Fall Prevention: Analysis and Visualization
Source: Int J Environ Res Public Health. 2022 Jul 7;19(14):8326. doi: 10.3390/ijerph19148326 (PMC9320470; doi:10.3390/ijerph19148326)
Supplement: Supplementary file 1 [file ijerph-19-08326-s001.zip › ijerph-1766526-supplementary.pdf]

Supplementary

Table S1. Table of the specific search process.

Set	Results	Topic	Set	Results	Topic
#1	2739	"Accidental Fall"	#12	2595	"Tai Chi"
#2	241954	"Fall"	#13	47	"Tai Ji Quan"
#3	2839	"Fall*, Accidental"	#14	8	"Chi, Tai"
#4	1930	"Fall and Slip"	#15	51	"Taichi"
#5	44400	"Fall Risk"	#16	53	"Taijiquan"
#6	46	"Risk*, Fall"	#17	368	"Tai Chi Chuan"
#7	14471	"Fall Prevention"	#18	4	"Ji Quan, Tai"
#8	10	"Prevention*, Fall"	#19	3	"Tai-yi"
#9	242109	#8 OR #7 OR #6 OR #5 OR #4 OR #3 OR #2 OR #1	#20	2875	#19 OR #18 OR #17 OR #16 OR #15 OR #14 OR #13 OR #12 OR #11 OR #10
#10	84	"Tai-ji"	#21	683	(#8 OR #7 OR #6 OR #5 OR #4 OR #3 OR #2 OR #1) AND (#19 OR #18 OR #17 OR #16 OR #15 OR #14 OR #13 OR #12 OR #11 OR #10)
#11	230	"Taiji"			

Table S2. Table of the Merged Keywords.

Set	Keywords	Merged keywords
1	tai chi	chuan, tai chi chuan, tai chi exercise, tai ji quan, chi, tai ji, taichi, ji quan
2	people	human
3	risk	falls risk, fall risk, risk factor
4	elderly	aging, older adult, aged, older person, elderly people, older people, elderly person, age, dwelling older adult, older, elderly adult, elderly faller
5	fall prevention	preventing fall, falls prevention, fall prevention, falls in the elderly, prevention
6	injury	injurious fall
7	trial	therapy

Table S3. Ranking of co-cited reference cluster.

Number	Silhouette	Name
0	0.752	One-leg stance
1	0.696	Aging population
2	0.847	People
3	0.825	Parkinsons disease
4	0.944	Muscle activation profile
5	0.801	Elderly-a review
6	0.758	Physical therapy
7	0.811	Physical function
8	0.929	Physical benefit
9	0.829	Abridgrd Cochrane
10	0.985	Vestibular rehabilitation
11	0.974	Parkinsons disease
12	0.995	Health outcome
13	0.97	Participant characteristics
14	0.98	Prevention service
15	0.986	Evidence show
16	0.914	Stair ascent
19	0.996	Exercise program
20	0.997	Care facilities
21	0.97	Health fitness professional

Table S4. The primary themes of Tai Chi fall prevention field.

Name	Theme
i	Targeted Therapy (Parkinson's disease, vestibular rehabilitation, fracture, frailty[1], osteoarthritis, osteoporosis, stroke)
ii	Community Fall Prevention (physical therapy, exercise program, home based exercise, quality of life, prevention service, care facilities, health fitness professional)
iii	Research Paradigm (one-leg stance, gait, balance control, postural control, proprioception, bone mineral density, strength[2], muscle activation profile, participant characteristics, aging population, elderly, women, evidence show, randomized controlled trial, meta-analysis, controlled trial, multifactorial intervention)
iv	Psychological Factors (cognitive impairment, dementia)

[1] The results of theme i include "frailty" and "disability", but since these two words are similar in meaning, we only present "failty" in the table.

[2] The results of theme iii include "strength" and "muscle strength", but since these two words are similar in meaning, we only present "strength" in the table.
